# The *RHCE* gene encodes the chicken blood system I

**DOI:** 10.1186/s12711-024-00911-9

**Published:** 2024-06-19

**Authors:** Janet E. Fulton, Amy M. McCarron, Ashlee R. Lund, Wioleta Drobik-Czwarno, Abigail Mullen, Anna Wolc, Joanna Szadkowska, Carl J. Schmidt, Robert L. Taylor

**Affiliations:** 1 Hy-Line International, Research and Development, PO Box 310, Dallas Center, IA USA; 2 Department of Animal Genetics and Conservation, Institute of Animal Science, Warsaw University of Life Sciences, Warsaw, Poland; 3 Department of Animal Science, Iowa State University, Ames, IA USA; 4 Department of Animal and Food Science, University of Delaware, Newark, DE USA; 5 Division of Animal and Nutritional Sciences, West Virginia University, Morgantown, WV USA

## Abstract

**Background:**

There are 13 known chicken blood systems, which were originally detected by agglutination of red blood cells by specific alloantisera. The genomic region or specific gene responsible has been identified for four of these systems (A, B, D and E). We determined the identity of the gene responsible for the chicken blood system I, using DNA from multiple birds with known chicken I blood system serology, 600K and 54K single nucleotide polymorphism (SNP) data, and lowpass sequence information.

**Results:**

The gene responsible for the chicken I blood system was identified as *RHCE*, which is also one of the genes responsible for the highly polymorphic human Rh blood group locus, for which maternal/fetal antigenic differences can result in fetal hemolytic anemia with fetal mortality. We identified 17 unique *RHCE* haplotypes in the chicken, with six haplotypes corresponding to known I system serological alleles. We also detected deletions in the *RHCE* gene that encompass more than 6000 bp and that are predicted to remove its last seven exons.

**Conclusions:**

*RHCE* is the gene responsible for the chicken I blood system. This is the fifth chicken blood system for which the responsible gene and gene variants are known. With rapid DNA-based testing now available, the impact of I blood system variation on response against disease, general immune function, and animal production can be investigated in greater detail.

**Supplementary Information:**

The online version contains supplementary material available at 10.1186/s12711-024-00911-9.

## Background

Blood groups or blood systems are due to red blood cell antigens that differ among individuals of the same species. These antigen differences were originally distinguished by agglutination, after mixing red blood cells with polyclonal antisera that detect specific variant(s). The first human blood group system (ABO) was discovered by Landsteiner in 1900, for which he received the 1930 Nobel Prize for Physiology or Medicine. The ABO blood system is the most immunogenic human blood system. Antigenic mismatch for this system results in severe and potentially lethal transfusion reactions. The second most significant human blood system is the highly polymorphic Rh blood system. Antigenic incompatibility for Rh between mother and fetus causes fetal hemolytic anemia, which can lead to fetal death. Correct identification of blood types has particular significance for human health, as these antigen differences are the bases for blood transfusion and tissue transplantation rejection. As of November 2023, the International Society of Blood Transfusion recognized 45 human blood systems that are genetically determined by 50 genes [1].

The human RH antigenic blood system is the most complex human blood system. It is encoded by two structural genes, *RHD* (Rh blood group D antigen) and *RHCE* (Rh blood group CcEe antigen), which are closely linked and have opposite reading frames on human chromosome 1. *RHD* is a paralog of *RHCE*, originating by duplication of the *RHCE* gene, and occurs only in the primate lineage [2, 3]. Both *RHD* and *RHCE* genes have 10 exons. The human Rh-negative phenotype is due to the deletion of the *RHD* gene. Expression of RHD and RHCE antigens on the surface of red cells requires RhAG antigen (Rh associated antigen), which is predicted to form a heterodimer with Rh proteins [2]. The Rh blood system has 56 recognized antigens, resulting from variants within either the RhD or RhCE proteins [4]. The antigenic difference between the Rhce and RhCe proteins is due to three transmembrane and one extracellular amino acid residues, whereas the antigenic difference between Rhce and RhcE proteins is due to one extracellular amino acid residue [5]. The specific functions of the Rh antigens are not known, although it is hypothesized that the protein encoded by the *RHD* gene is a transmembrane NH_3_ transporter, while the protein encoded by the *RHCE* gene plays a role in CO_2_ transport [6–8].

The existence of blood systems in the chicken was first reported by Landsteiner and Miller [9]. Extensive work by Briles and Gilmour subsequently identified 13 blood systems in the chicken [4, 10–12]. These systems, discovered by red blood cell agglutination with polyclonal sera, were named in alphabetical order of discovery, and include A, B, C, D, E, H, I, J, K, L, N, P and R [13]. Early work with chicken blood systems focused on their potential as genetic markers for specific traits, especially production traits, such as egg number or body weight, and as markers for disease resistance or immune-related traits. The relevant gene or genomic region responsible for four of these blood systems has been identified. The B system identifies variation in the chicken major histocompatibility complex (MHC), which has a major role in transplantation rejection [14]. In addition, its significant impact on responses to multiple bacterial, viral, and parasitic pathogens [15] identifies the chicken B system as the best animal model for MHC-associated disease resistance [16]. Recent studies have revealed that the closely-linked blood systems A and E are encoded by, respectively, the *C4BPM* gene (*complement component 4 binding protein, membrane*), a member of the regulation of complement activation gene cluster, and by the nearby *FCAMR* gene (*Fc alpha and mu receptor*) [17]. The chicken D blood system results from variation in the *CD99* (*cluster of differentiation 99*) gene [18]. In humans, the proteins encoded by the *CD99* gene and the paralogous *Xg* (*Xg blood group*) gene, define the Xg^a^/CD99 blood group [19].

The chicken alloantigen system I was discovered by Briles [11], with eight alleles, I^1^ through I^8^, identified. Direct or indirect effects of the I system on several traits have been documented. Egg size had some relationship with the I system, as I^4^ was present in large egg lines, while I^2^ was not and, conversely, I^2^ was present in small egg lines but not I^4^ [20]. In an experiment that tested multiple alloantigen systems in the context of two different B-complex backgrounds, birds with the I^8^I^8^ genotype had higher macrophage nitrite production compared to B^19^B^19^ or B^19^B^21^ birds with I^2^I^8^ or I^2^I^2^ genotypes, respectively (Qureshi et al., personal communication). When the B genotype was B^2^B^2^ or B^2^B^5^, the I^8^I^8^ genotype resulted in more macrophage IL-6 than the I^2^I^8^ or I^2^I^2^ genotypes. Selection for bursa of Fabricius size altered the I allele frequency, with the large bursa line having a 91% frequency of the I^2^ allele, compared to 45% for the small bursa line [21]. In a line selected for high antibody response to sheep red blood cells, I^4^I^4^ individuals had a greater response against cecal coccidiosis than I^2^I^2^ or I^2^I^4^ individuals [22].

Using classical recombination studies and chromosomal translocations, pea comb and the I blood system were mapped to chromosome 1, separated by 32.9 centi-Morgan (cM) [23]. Pea comb is now known to be due to variation in the *SOX5* gene on chromosome 1 (at 65.4 Mb) confirming the chromosomal location of the causative gene [24, 25]. Later studies showed independent segregation of pea comb and the I blood system phenotypes, thus the chromosomal location of the gene for the I system remains unknown [26].

Precise serological identification of blood system alleles can be difficult due to the complex nature of both polyclonal antisera and red blood cell antigens that determine blood types. Polyclonal antisera contain multiple antibodies that react against many epitopes. Because of the diverse antibody repertoire, polyclonal antisera can frequently cross-react with multiple antigens [27, 28]. Adsorption can remove some of these cross-reactive antibodies, but the process is time-consuming and can be imprecise. Accurate, consistent identification of serological alleles requires the use of multiple antisera, as well as exchange of both polyclonal antisera and antigenic red cells among laboratories to ensure consistency [27, 29]. This comparison testing has aided standard reactivity and nomenclature for the chicken MHC-B serological typing. Interlaboratory testing of antibodies or antigens has not been done for any other chicken blood systems. Serological typing for chicken blood types on different lines was done 50–60 years ago [4, 30] but typing reagents for these chicken blood systems are no longer available to repeat and confirm previous typing results.

The objective of this study was to identify the genetic region that encodes the chicken I blood system, with the final goal being the development of DNA detection-based methods for the identification of I system alleles to allow further studies on the impact of this blood system on phenotypic traits. Multiple samples were available from independent lines that had I blood system serological information. In addition, sequences were available from inbred research lines for which the I system alleles were known. Our preliminary work indicated that the chicken I blood system was encoded by variation within a region that included the chicken RHCE gene. This led to a detailed exploration of the chicken RHCE gene and identification of gene variants responsible for specific I blood system serologically-defined alleles. In addition, birds carrying a homozygous deletion encompassing exons 3–10 of the chicken *RHCE* gene were identified.

## Methods

### Genetic material

DNA was available from many individuals from different lines for which I blood group alleles had been previously determined. All serological typing of Hy-Line (HYL) samples had been performed at the Northern Illinois University (NIU) laboratory between 2000 and 2008 by Ruth and Elwood Briles (Hy-Line, unpublished). The lines included three elite white egg lines (White Leghorn breed, WL) from Hy-Line International (WL1, WL2, WL7), which were identified to be segregating for alleles I^2^ and I^8^. Additional DNA samples from three elite white egg lines (WL3, WL6, WL9) and three elite brown egg lines of two breeds (Rhode Island Red; RIR1 and White Plymouth Rock, WPR1 and WPR2), for which no I system serological information was known, were also available. Samples were also obtained from the NIU DNA bank, which consists of approximately 2500 DNA samples from individuals typed for multiple alloantigen systems using alloantigen specific antisera. Some samples were collected from progeny of pedigreed matings, while other samples had no pedigree information. For this study, we obtained 40 samples from one pedigreed family that was known to be segregating for the I^2^ and I^8^ alleles, and 88 samples with no pedigree information but that were known to carry the I^2^ and/or I^8^ alleles (Table 1). DNA was also available from three inbred lines: UCD001 which is the line used for the original chicken genome reference (builds 2–6), UCD003, and ADOL-15I_5_. Genome sequence information from inbred lines was also available, as listed in Table 1, and their I system allele information was obtained from the literature [31–33].

**Table 1:** Genetic resources and analysis methods used to identify the I system gene and to define chicken blood system I alleles

Source	Breed	Number of samples	I alleles	Method					References
				DNA^a^	Genomic sequence^b^	4 × sequence^c^	GWAS 600k^d^	GWAS 54k^e^	
NIU DNA bank (pedigree)	WL, Ancona	40	I^2^, I^8^	X	nd	X	P	nd	None
NIU DNA bank (non-pedigree)	WL, Ancona	88	I^2^, I^8^	X	nd	X	P	nd	None
UCD001	RJF	11	unk	X	X	nd	nd	nd	[29]
UCD003	WL	16	I^8^	X	X	nd	nd	nd	[27, 28]
ADOL-15I_5_	WL	3	I^8^	X	X	nd	nd	nd	[27, 30]
WL1	WL	89	I^2^, I^8^	X	nd	X	P	X	None
WL2	WL	79	I^2^, I^8^	X	nd	X	P	X	None
WL3	WL	46	unk	X	nd	nd	nd	nd	None
WL6	WL	140	unk	X	nd	nd	nd	nd	None
WL7	WL	34	I^2^, I^8^	X	nd	X	P	nd	None
WL9	WL	272	unk, fixed	X	nd	nd	nd	nd	None
WPR1	WPR	46	unk	X	nd	nd	nd	nd	None
WPR2	WPR	46	unk	X	nd	nd	nd	nd	None
RIR1	RIR	60	unk	X	nd	X	nd	nd	None
ADOL 6_1_	WL	seq	I^2^	nd	X	nd	nd	nd	[27, 30]
ADOL 7_2_	WL	seq	I^3^	nd	X	nd	nd	nd	[27, 30]
RHC	WL	seq	I^4^	nd	X	nd	nd	nd	[27, 30]
Wellcome	WL	seq	I^2^	nd	X	nd	nd	nd	[27]
ADOL-15I	WL	seq	I^8^	nd	X	nd	nd	nd	[27, 30]

*RIR* Rhode Island Red, *WL* White Leghorn, *WPR* White Plymouth Rock, *GWAS* genome-wide association study, *unk* unknown, *nd* = no data, *seq* publicly available genome sequence, *X* data obtained, *P* pooled DNA, *Ref* reference citations

^a^Individual DNA available

^b^Public genomic sequence

^c^Multiple individual DNA sequences

^d^Pooled DNA used for GWAS

^e^individual DNA used for GWAS

Five sets of DNA pools were made from different genetic sources, as indicated in Table 1, with each set consisting of three pools of DNA from birds that were serologically identified as being I^2^I^2^, I^2^I^8^, or I^8^I^8^. The number of samples within each pool ranged from 4 to 15 (184 samples, in total), with equivalent amounts of DNA from each sample used to contribute to a pool. The sample sources were serotyped progeny from NIU pedigree families for which both parents were known to be I^2^I^8^ heterozygotes, non-pedigree individuals from NIU with known I alleles, and individuals with known serological alleles from three HYL lines (WL1, WL2, WL7) that were segregating for I system alleles.

Different analysis methods were used for different sample sets, as summarized in Table 1, depending on availability of samples, genomic resources, and serological information.

### Genome-wide association studies (GWAS)

The Affymetrix Axiom 600K chicken SNP array was used to genotype the five sets of DNA pools. Genotyping was performed by GeneSeek (Lincoln, NE, USA) and Affymetrix Analysis Power tools were used for subsequent analysis. A 0.1 threshold was used as the minimum minor allele frequency (MAF) and the maximum missing genotypes for inclusion in the analyses. In total, 580,961 single nucleotide polymorphisms (SNPs) were available for analysis after quality control.

A custom R script was used for analysis of the intensity files from the 600K SNP Affymetrix chip. The number of copies of the B allele for each sample was recoded as 0 for AA, 1 for AB, and 2 for BB genotypes. A regression equation was developed for each SNP, where the expected allele count (based on serology information) was used as the response variable and intensity was used as the explanatory variable. The coefficient of determination (*R*^2^) and p-value were calculated from the regression analysis and used to identify SNPs that segregated in accordance with expected pool genotypes. The region on microchromosome 23 with the highest *R*^2^ value was subsequently scanned for genes predicted to encode for proteins with cell membrane expression.

Individual genotypes were obtained from DNA of birds with known I blood system serology using a 54K Axiom chicken SNP array, including 50 individuals from the WL1 line, of which 31 were I^2^I^2^ (case), and 19 were I^8^I^8^ or I^2^I^8^ (control), and 53 individuals from the WL2 line, of which 31 were I^2^I^2^ (case) and 22 individuals were I^8^I^8^ or I^2^I^8^ (control). The 54K SNP array consists of a subset of the 600K Axiom SNP array set. These individuals were different than those used for the DNA pools with 600K SNP information. In total, 49,760 SNPs were available for the analysis, after applying a MAF filter of at least 0.05 and a minimum call rate per SNP of 0.99. Use of individual samples on the 54K SNP array rather than DNA pools allowed lowering of the MAF threshold relative to the pools analysis and a more stringent call rate threshold to retain only high-quality SNPs. Case/control (as defined above) association analysis was then carried out separately for each line in order to exclude random results that could result from the genetic characteristics of individual breeding lines and small sample sizes. Manhattan-type plots were created using the qqman library in the R programming language. The Bonferroni correction significance threshold of negative log_10_ (p value) = 6.000512 was determined using the number of independently segregating markers.

### Sequence analysis

Low-pass sequence information was also obtained for individual samples with known I system alleles. Each sample was low-pass (1 ×) sequenced four times and, then, the four bam files were combined to produce a 4 × coverage sequence file. Sequence information for DNA samples with the same I system serological phenotype (I^2^I^2^, I^2^I^8^, or I^8^I^8^) were compared within sample source (HYL line or NIU DNA bank). Comparison of I^2^I^2^, I^2^I^8^, and I^8^I^8^ samples (n = 4, 7, and 17, respectively) identified the same region as identified based on the DNA pools with 600K genotypes, but with a weaker signal, which is likely due to the small sample size. Additional low-pass (4 ×) genome sequences were obtained from 60 RIR1 (with no serological information) for detailed sequence examination.

### Identification of candidate genes

The dataset contained complete genomic sequences from samples of WL1 with known serological types (I^2^I^2^ = 3, I^2^I^8^ = 8, I^8^I^8^ = 10; different from those used for DNA pooling or 54K genotypes). Pre-processing, including GRCg7b reference genome mapping and quality control, and joined variant calling, resulted in a vcf file. BWA and a standard GATK pipeline were used to align sequences and to call variants. The program SNPEff was used for variant annotation, while the program SNPSift identified variants with HIGH and MODERATE impact on protein function according to sequence ontology terms. Bioinformatic analysis was performed using BWA, Plink v.1.9 beta [34], SnpEff v5.0e [35], SnpSift v5.0e [36], GATK v4.2.3.0 [37], R v.4.1.1 [38], and qqman library [39].

Analysis of complete genomic sequences provided information on all variants that may impact blood system I. With a focus on the region identified in the GWAS, SNP and indel variants were analyzed for the I^2^I^2^ and I^8^I^8^ serologically determined phenotypes.

The Uniprot and NCBI databases were used to select candidate genes for the chicken blood system I. Particular attention was paid to genes that encode proteins that are present in the outer cell membrane (GO:0005886). Selected genes had to be in the region identified in the GWAS (on microchromosome 23) and have SNPs predicted to have a medium (MODERATE) or high (HIGH) effect on protein function, as defined by gene annotation. SNP variant frequency in the region was examined by comparing opposing homozygote groups of samples. The frequency had to be equal or greater than 0.9 in the group that was serologically identified as homozygous I^2^I^2^ or I^8^I^8^, and 0 in the other group.

### SNP genotyping and identification of haplotypes

Build 6 (GRCg6a) of the chicken reference genome was used to identify SNPs within the candidate region. Only SNP alleles that were present in the previously obtained HYL sequences and predicted to impact the amino acid encoded by the candidate gene were used [40]. SNP detection was done using allele-specific fluorescence detection with PACE^®^ chemistry (3CR Bioscience Ltd., Harlow, UK), which uses one common and two allele-specific primers, and is capable of identifying both specific SNP alleles and the presence or absence of insertion/deletions [41]. A gene specific SNP panel containing 15 SNPs was developed for genotyping the top candidate gene. Limited combinations of SNP alleles (haplotypes) were found and each haplotype was assigned a number based on the order of its identification, except that RHCE-H02 was assigned to I^2^. All SNPs for which validated assays could be developed are listed in Table 2. This table also includes the SNP genomic information, gene location, and putative codon and nucleotide changes. The reference allele was defined as the allele given in the RJF reference sequence (build 6), with the alternative allele being the one found in the HYL samples. It should be noted that DNA from the actual reference genome was available and this was heterozygous for multiple SNPs.

**Table 2:** SNP name and genomic location of the SNPs used to define the *RHCE* gene haplotypes

SNP	Chr 23:bp	Location in the gene	Codon change	aa change	Ref. > Alt	RHCE haplotypes					
						H01	H02	H03	H04	H05	
rs740623580	2,749,185	Exon 1	CGA > CAA	R9Q	G > A	G	G	G	G	A	
rs869007872	2,749,241	Exon 1	TCT > TTT	S27F	C > T	C	T	T	C	C	
rs794503708	2,749,275	Exon 1	CCC > CTC	P39L	C > T	C	T	T	C	C	
rs733753324	2,749,792	Exon 2	TTG > TTC	L43F	G > C	G	C	G	G	G	
rs731472668	2,749,926	Exon 2	CAT > CGT	H88R	A > G	A	G	A	A	A	
rs738943348	2,749,941	Exon 2	CTG > CCG	L93P	T > C	T	C	T	T	T	
rs739602946	2,749,952	Exon 2	TCA > CCA	S97P	T > C	T	C	T	T	T	
rs738898886	2,750,212	Exon 3	ATA > ATG	I101M	A > G	A	G	A	A	A	
rs313465722	2,750,348	Exon 3	TAT > CAT	Y147H	T > C	T	C	T	T	T	
rs735870559	2,751,919	Exon 5	GTG > TTG	V215L	G > T	G	G	G	G	G	
rs316593393	2,752,402	Exon 6	AAG > GAG	K272E	A > G	A	A	G	G	G	
rs733511284	2,752,431	Exon 6	GGT > GGG	G281G	T > G	T	T	G	G	G	
rs314800215	2,752,791	Exon 7	GAG > AAG	E325K	G > A	G	A	G	G	G	
rs738163839	2,752,814	Exon 7	GAC > GAA	D332E	C > A	C	A	C	C	C	
rs737604974	2,753,525	Exon 8	GAG > AAG	E343K	G > A	G	A	G	G	G	
I system serological allele						I^8^	I^2^	I^4^, I^8^	I^8^		
Line source						NIU	Line 6_1_	UCD003	NIU	WPR1	
						^a^UCD001	NIU	RHC	WPR1	WPR2	
						WL1	WL1 WL2	WL2	WPR2		
						WL3	WL3	WL3			
						WL6	WL6	WL6			
						WL7	WL7				

**SNP**	**Chr 23:bp**	**Location in the gene**	**Codon change**	**aa change**	**Ref. > Alt**	**RHCE haplotypes**					
						**H06**	**H07**	**H08**	**H10**	**H11**	
rs740623580	2,749,185	Exon 1	CGA > CAA	R9Q	G > A	G	G	G	G	G	
rs869007872	2,749,241	Exon 1	TCT > TTT	S27F	C > T	C	C	T	C	C	
rs794503708	2,749,275	Exon 1	CCC > CTC	P39L	C > T	C	C	T	C	C	
rs733753324	2,749,792	Exon 2	TTG > TTC	L43F	G > C	G	G	G	G	G	
rs731472668	2,749,926	Exon 2	CAT > CGT	H88R	A > G	A	A	G	A	A	
rs738943348	2,749,941	Exon 2	CTG > CCG	L93P	T > C	T	T	C	T	T	
rs739602946	2,749,952	Exon 2	TCA > CCA	S97P	T > C	T	T	C	T	T	
rs738898886	2,750,212	Exon 3	ATA > ATG	I101M	A > G	A	–	G	A	A	
rs313465722	2,750,348	Exon 3	TAT > CAT	Y147H	T > C	T	–	C	T	T	
rs735870559	2,751,919	Exon 5	GTG > TTG	V215L	G > T	G	–	T	G	G	
rs316593393	2,752,402	Exon 6	AAG > GAG	K272E	A > G	G	–	G	G	A	
rs733511284	2,752,431	Exon 6	GGT > GGG	G281G	T > G	G	–	G	G	T	
rs314800215	2,752,791	Exon 7	GAG > AAG	E325K	G > A	A	–	G	G	A	
rs738163839	2,752,814	Exon 7	GAC > GAA	D332E	C > A	A	–	C	C	A	
rs737604974	2,753,525	Exon 8	GAG > AAG	E343K	G > A	A	–	G	A	G	
I system serological allele									I^8^	I^3^	
Line source						^a^UCD001	RIR	RIR	ADOL-15I5	Line 7_2_	
								WPR1	NIU	WL2	

**SNP**	**Chr 23:bp**	**Location in the gene**	**Codon change**	**aa change**	**Ref > Alt**	**RHCE haplotypes**					
						**H12**	**H13**	**H14**	**H15**	**H16**	**H17**
rs740623580	2,749,185	Exon 1	CGA > CAA	R9Q	G > A	G	G	G	G	G	G
rs869007872	2,749,241	Exon 1	TCT > TTT	S27F	C > T	T	T	C	T	C	T
rs794503708	2,749,275	Exon 1	CCC > CTC	P39L	C > T	T	T	C	T	C	T
rs733753324	2,749,792	Exon 2	TTG > TTC	L43F	G > C	C	G	G	C	G	G
rs731472668	2,749,926	Exon 2	CAT > CGT	H88R	A > G	G	A	G	G	A	A
rs738943348	2,749,941	Exon 2	CTG > CCG	L93P	T > C	C	T	C	C	T	T
rs739602946	2,749,952	Exon 2	TCA > CCA	S97P	T > C	C	T	C	C	T	T
rs738898886	2,750,212	Exon 3	ATA > ATG	I101M	A > G	–	–	A	G	–	A
rs313465722	2,750,348	Exon 3	TAT > CAT	Y147H	T > C	–	–	T	C	–	T
rs735870559	2,751,919	Exon 5	GTG > TTG	V215L	G > T	–	–	G	T	–	G
rs316593393	2,752,402	Exon 6	AAG > GAG	K272E	A > G	–	–	G	G	–	G
rs733511284	2,752,431	Exon 6	GGT > GGG	G281G	T > G	–	–	G	G	–	G
rs314800215	2,752,791	Exon 7	GAG > AAG	E325K	G > A	–	–	G	G	–	G
rs738163839	2,752,814	Exon 7	GAC > GAA	D332E	C > A	–	–	C	C	–	C
rs737604974	2,753,525	Exon 8	GAG > AAG	E343K	G > A	–	–	G	G	–	A
I system serological allele											
Line source						WL2	WL2	WPR1	WPR1	WL1	WL6
						WL6	WL6			WL6	

Also provided are codon information and predicted amino acid change based on chicken build GalGal1b.mat.broiler.GRCg7b. The I blood system allele found for each haplotype is listed when known and the specific line source of each haplotype is indicated

–: Failure of PCR to provide result, Ref.: reference allele; Alt: alternate allele

^a^UCD001 reference genome (GRCg6a) is heterozygous for RHCE- H01 and RHCE-H06

### Genome and protein comparative analyses

Chicken and human RHCE chromosomal regions were visualized using the NIH Comparative Genome Viewer [42] to align chicken microchromosome 23 (build:GalGal1b.mat.broiler.GRCg7b) with human chromosome 1 (build:GRCh38.p14). The position of the deletion that affects the chicken RHCE region was identified using low-pass genome sequence bam files aligned with the *Gallus gallus* genomic sequence** (**GalGal1b.mat.broiler.GRCg7b) using the IGV browser [43]. Protein structural predictions were obtained using AlphaFold [44, 45] and PredictProtein [46].

## Results

### Genome-wide association studies

**Fig. 1:**
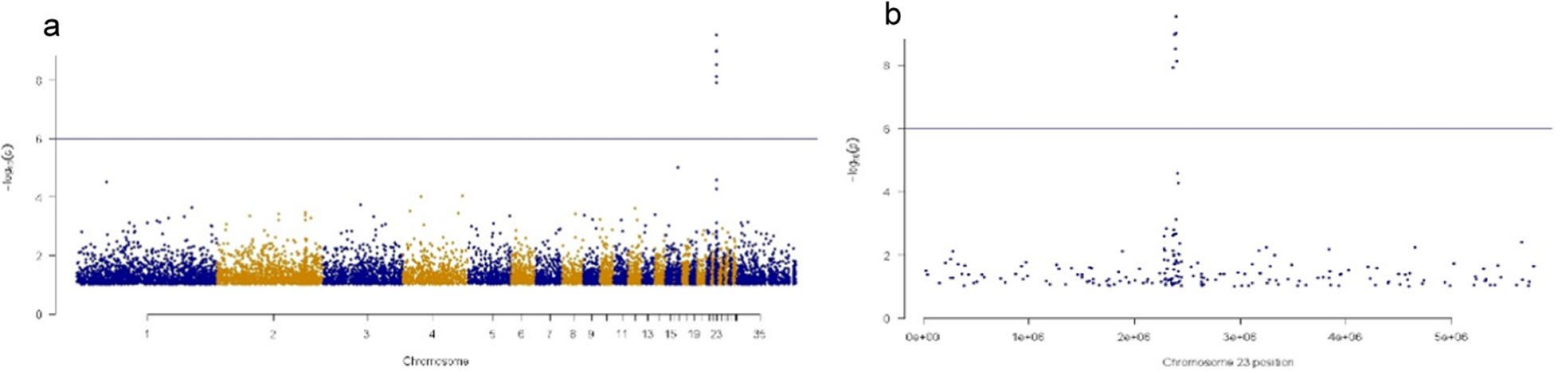
GWAS results generated from 600K SNP genotypes and five sets of three DNA pools containing either I^2^I^2^, I^2^I^8^ or I^8^I^8^ serologically defined phenotypes. **a** Whole-genome GWAS; **b** Microchromosome 23 GWAS

The GWAS of the five sets of DNA pools with 600K SNP genotypes showed one very strong peak on microchromosome 23 (p = 3.355E−10) (Fig. 1a), between 2.2 and 2.4 Mb (Fig. 1b) (build 6). The additional GWAS on different samples and low-pass sequences (1 ×, 4 times) confirmed the same region on microchromosome 23, although the signal was weaker, which is most likely due to the smaller sample size (data not shown). All subsequent analyses focused on this 200,000-bp region.

### Identification of candidate genes

Based on the low coverage sequence analysis of line WL1, seven SNP with alleles that matched expected frequencies (SNP I^2^I^2^ FA $\ge$ 0.9 and I^8^I^8^ FA = 0) were identified on microchromosome 23, located near or within four genes: *MAP3K6*, *RAB42*, *RHCE*, and *LOC107055024*. Based on the Uniprot and NCBI databases, the products of these genes were assigned molecular and biological functions, as well as their predicted cellular location. The products of two of the genes, *RHCE* and *LOC107055024*, are predicted to be in the outer cell membrane. Sequence analysis identified four non-synonymous SNPs within the *RHCE* gene and one SNP within *LOC107055024*. Only the SNP within the *RHCE* gene agreed with the expected I blood system variation within different lines, which is consistent with the RHCE SNP association found with specific individuals and their chicken I blood system serology.

### Haplotype definitions

Samples from multiple lines (some with known serology) were genotyped with the 15-SNP assay set that was developed to define the *RHCE* gene haplotypes. Table 2 summarizes these SNPs, including their location (based on build 7b), the position within exon, codon change, predicted amino acid change, the 17 unique haplotypes with, when known, the I system serological allele associated with each one, and the specific lines that contained the various haplotypes.

Line WL1 was previously known to contain two serological I system alleles (I^2^ and I^8^) and we found two RHCE haplotypes (RHCE-H01 and RHCE-H02). All 19 I^2^I^2^ samples were homozygous for RHCE-H02, indicating that I^2^ is RHCE-H02. Of the 28 samples identified as I^8^/I^8^, 14 were homozygous for RHCE-H01, and 14 were heterozygous RHCE-H01/RHCE-H02. Forty-two samples were identified as I^2^I^8^ and of these, 40 were RHCE-H01/RHCE-H02 heterozygotes and two were RHCE-H01 homozygotes. Although agreement is not perfect, this suggests that I^8^ is RHCE-H01. Within this line, there was a discrepancy between I allele and RHCE haplotype for 16 of the samples, and for 14 of these, correct identification of the homozygous I^8^I^8^ individuals based on serological information failed. Correspondence between serology and SNP haplotype was 82%, with most of the errors due to failure to distinguish I^8^I^8^ homozygotes from heterozygotes based on serology.

Line WL2 also had two I system serological alleles, I^2^ and I^8^, and two RHCE haplotypes (RHCE-H02 and RHCE-H03). The same RHCE-H02 haplotype as identified in WL1 was homozygous for all 29 I^2^I^2^ homozygotes in WL2. The second haplotype (RHCE-H03) was homozygous in six of the seven I^8^I^8^ homozygotes. Of the 43 samples identified as I^2^I^8^, 38 were heterozygous for RHCE-H01 and RHCE-H03 and five were homozygous for RHCE-H03. These results are consistent with I^8^ being RHCE-H03. Correspondence between serology and SNP haplotype was 92%, with the failure to identify I^8^ homozygotes representing the largest source of error.

Line WL7 had two I system alleles (I^2^ and I^8^) and two RHCE haplotypes (RHCE-H01 and RHCE-H02). There were four I^2^I^2^ homozygotes, which were all RHCE-H02 homozygotes, and all 15 I^8^I^8^ homozygotes were homozygous for RHCE-H01. This confirms that I^2^ is RHCE-H02 and I^8^ is RHCE-H01. Within the 43 samples identified as I^2^I^8^, 32 were RHCE-H01/RHCE-H02 heterozygotes and 11 were RHCE-H01/RHCE-H01 homozygotes. Discrepancies concerned only failure to distinguish between I^8^ heterozygotes and homozygotes, and the overall error rate was 12%.

Line WL9 was known to be fixed for one I system allele, although the specific allele is not known (internal Hy-Line unpublished report, 2002). RHCE SNP genotyping of 242 samples from that line confirmed the presence of only one RHCE SNP haplotype (RHCE-H03), which is consistent with the previous serology report of I blood system homozygosity.

Thus, four very distinct WL lines showed consistency between the number of serologically identified alleles and the number of RHCE haplotypes found. Furthermore, the three lines in which the serologically defined I^2^ allele segregated, carried the RHCE-H02 haplotype, while I^8^ was associated with either RHCE-H01 in two of the lines and RHCE-H03 in the third.

From the NIU DNA bank, the 40 individuals produced from known I^2^ and I^8^ segregating families carried three haplotypes. All 21 I^2^I^2^ homozygotes were RHCE-H02 homozygotes, five of the I^8^I^8^ homozygotes were RHCE-H04 homozygotes, one I^8^I^8^ homozygote was a RHCE-H03 homozygote and three were heterozygotes, with RHCE-H02 and either RHCE-H03 or RHCE-H04. For the 88 non-pedigree NIU DNA bank samples, 15 of the 16 I^2^I^2^ were RHCE-H02 homozygotes and 38 of the 40 I^8^I^8^ were RHCE-H03 homozygotes. The observation that I^8^ was associated with both RHCE-H03 and RHCE-H04 within samples from the same laboratory (NIU) confirms the previous observation that these two RHCE haplotypes can be identified by the same serological reagent. Also, as found for other lines, I^2^ is RHCE-H02. Again, failure to identify heterozygotes and I^8^ homozygotes was the largest source of inconsistency. The overall consistency rate between serological identification and RHCE-SNP haplotype was 90% for both NIU sample sets. A summary of consistency across all five lines with I system segregation is provided in Additional file 1: Table S1; overall 88/89 (99% accuracy) of the serological I^2^I^2^ individuals were RHCE-H02 homozygotes, 76/99 (77% accuracy) of the serological I^8^I^8^ individuals were homozygotes for either RHCE-H01, RHCE-H03, and/or RHCE-H04, and 124/142 heterozygotes were identified (87% accuracy).

The availability of genome sequence information from inbred lines provided further confirmation of the relationship between RHCE haplotypes and I blood system serological alleles. Line UCD003 had previously been reported to carry I^8^ [31, 32] and here we found that it carried the RHCE-H03 haplotype. Roslin line 6_1_ was reported to carry I^2^ [31, 32] and was shown here to carry the RHCE-H02 haplotype. Line RHC was reported to carry I^4^ and was shown here to carry the RHCE-H03 haplotype. Thus, I^4^ is RHCE-H03, although we could not confirm this from a different sample source. It should be noted that line RHC originated in the UK, while the majority of the other lines are from the US, thus it is possible that they were not typed within the same laboratory with identical reagents. Line 15I_5_ has been reported to carry I^8^ [31, 32, 47] and was shown here to carry a unique haplotype, RHCE-H10, which differs from RHCE-H04 (also I^8^) by only the last SNP (rs737604974; exon 8). Roslin line 7_2_ was reported to carry I^3^ [31, 47] and was shown here to carry the unique haplotype RHCE-H11.

The RHCE SNP genotype results from the sample of the UCD001 reference genome were unexpected, as this DNA was from the same bird that was used to produce the original RJF reference (builds 2–6) and was heterozygous for many of the RHCE SNPs. Ten additional DNA samples were available from the same UCD001 inbred line (courtesy of Marcia Miller, City of Hope, Duarte, CA). The same heterozygous SNP pattern was detected in six of these samples and the remaining four were homozygous for a novel haplotype that was assigned the name RHCE-H06. The heterozygous samples (including the reference sample) were determined to be RHCE-H02/RHCE-H06.

Thus, results based on multiple sources and the use of independent lines, show consistency in the assignment of specific RHCE haplotypes to serological I alleles. RHCE-H02 is found for the I^2^ allele for seven chicken lines, including Roslin line 6_1_. Results based on three independent sources, including the UCD-003 line, show that RHCE-H03 is found for the I^8^ allele. Both RHCE-H03 and RHCE-H04 were found to define the I^8^ allele in the NIU DNA bank, which indicates that the I^8^ reagent did not distinguish between these two haplotypes.

Examination of the four haplotypes that result in I^8^ showed a consistent cluster of six variants, all of which differ from the I^2^ allele. Furthermore, examination of the predicted 3D protein structure of chicken RHCE indicated that these amino acids were located on the exterior of the protein, near each other. These six variants are L43F through Y147H and are all encoded by exons 2 and 3. It is likely that this is the epitope that distinguishes between the two serologically defined alleles, I^8^ and I^2^.

**Fig. 2:**
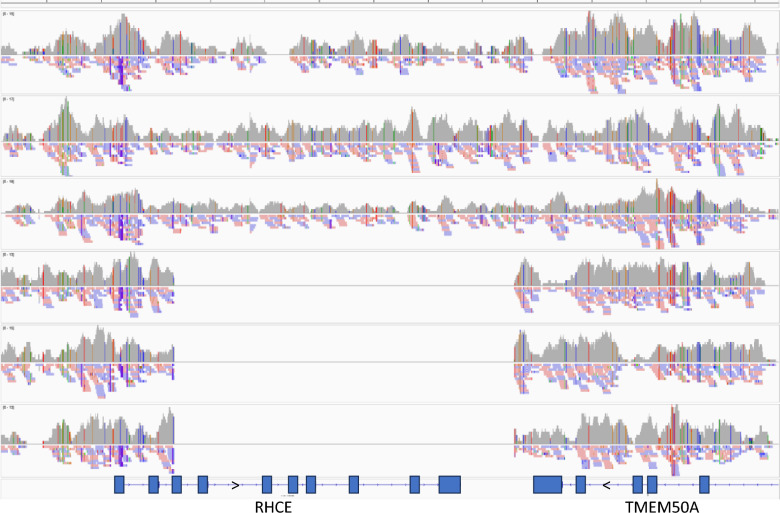
Alignment of sequences from three wild type and three ‘short’ haplotypes showing the extent of the deletion in the *RHCE* gene and the consistency of end and start locations between multiple samples. The region shown covers the *RHCE* gene and a portion of the *TMEM50A* gene

Additional HYL samples, including samples from different breeds and lines (WL3, RIR1, WPR1, WPR2), were used to determine whether other RHCE haplotypes existed, although no serological information was available for any of these samples. Multiple novel haplotypes were found, including four for which the last nine SNPs failed to produce a PCR product, suggesting that this part of the gene (end of exon 3 to end of gene) is missing (RHCE-H07, 12, 13, 16). It should be mentioned that the presence of these ‘short’ haplotypes can only be distinguished with the RHCE SNP panel if they are in the homozygous state. Examination of 4 × genome sequences from the RIR1 line, which had the highest frequency of these ‘short’ haplotypes, showed that individuals that carried this haplotype clearly lacked the region encompassing microchromosome 23: 2,539,342–2,545,584 (build 6), which includes the last 43 codons of exon 3 through to the end of the RHCE gene, i.e. a 6243-bp deletion (Fig. 2). The successful development of a PACE-based assay that detects the specific sequences defined by the deletion validated that the deletion occurs at the location indicated (data not shown).

### Protein model

**Fig. 3:**
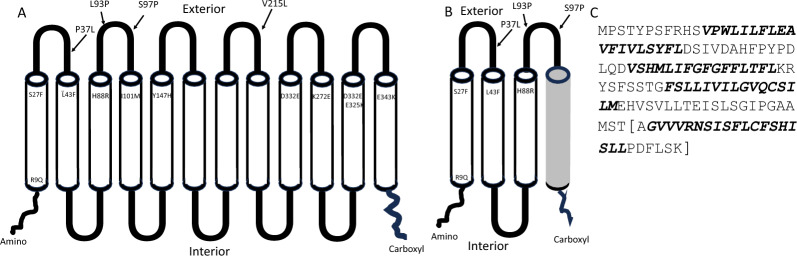
Cartoon depicting (**a**) Full length *RHCE* protein as predicted by AlphaFold; **b** Predicted topology of the protein encoded by the gene with the deletion; and (**c**) Predicted amino acid sequence of the protein encoded by the gene with the deletion. Variants are indicated based on their amino acid position. Four are in the loops between the transmembrane domains and 11 are within the alpha helices predicted to form the transmembrane domains. The dark gray region (in **b**) depicting the 4th transmembrane domain is derived downstream of the deletion endpoint which is proximal to the 3′ end of the TMEM50A gene. Transmembrane domains (in **c**) are depicted in italics. The sequence in the brackets is derived from the fusion created by the deletion

The predicted structure of the chicken RHCE protein is shown in Fig. 3. It is composed of 12 transmembrane alpha helices, with loops extending to the intra- and extracellular region, similar to the human RHCE protein. The amino acid variants identified by the RHCE SNP panel are shown in Fig. 3a. All but one of the 14 amino acid substitution variants are located in the extracellular region of the RHCE protein, on either a loop or on the end of an alpha helix. The six amino acid variants that distinguish I^2^ from I^8^ (L43F through Y147H) are clustered in the region within or near to the exterior of the cell membrane, providing a large epitope region available to elicit antibody production. The deletion variant, described above, encodes alpha helices one through three (Fig. 3b). The amino acid sequence acquired after the deletion is predicted to complete a fourth transmembrane alpha helix, with the last six amino acids being intracellular (Fig. 3c).

### Syntenic region

**Fig. 4:**
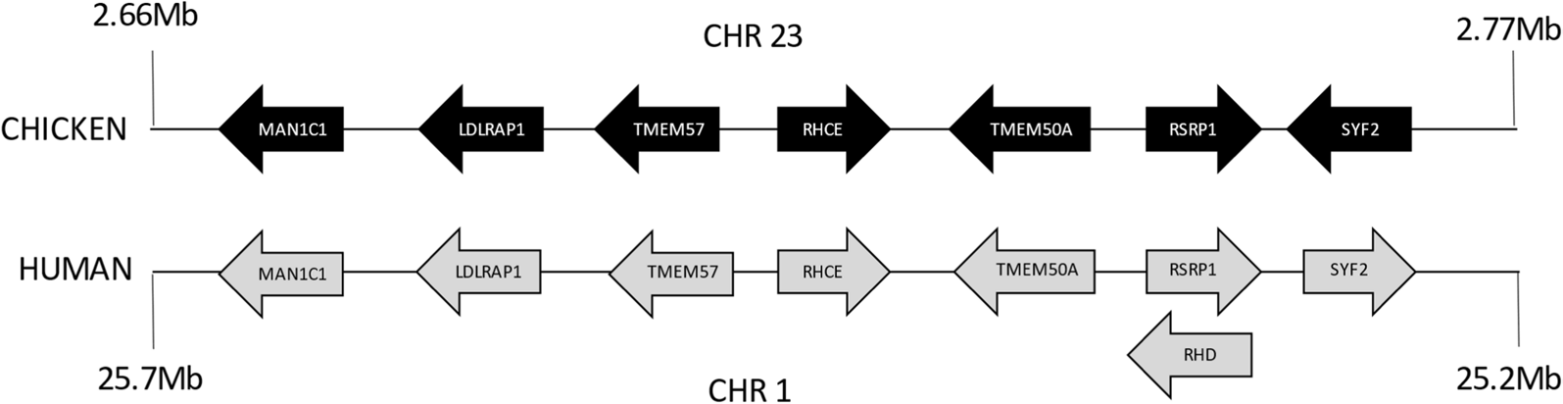
Comparative genome map of the chicken (GalGal1b.mat.broiler.GRCg7b) RH region with the human (GRCh38.p14) syntenic region. This map is not to scale

The *RHCE* gene is located on the chicken microchromosome 23, which is 6.1 Mb long. Comparison with the human genome (GRCh38.p14) indicates that most of the chicken microchromosome 23 is syntenic with a region on the short arm of human chromosome 1, except for one small segment that is syntenic with human chromosome 6. Figure 4 shows an alignment of the chicken and human genome regions that contain the *RHCE* gene plus three genes on either side of *RHCE*. All these genes are in the same order and orientation in the chicken and human genomes, except for *SYF2*, which is in the opposite orientation. The *RHD* gene is present only in the primate lineage and overlaps with the *RSRP1* gene in humans. No region of similarity with *RHCE* is found within the chicken *RSRP1* gene, which supports the absence of the *RHD* gene in the chicken genome. The chicken RHCE protein shows equivalent identity (cDNA and protein) and similarity (protein) to the human RHCE and RHD proteins, which makes it difficult to establish orthology based on sequences. However, the syntenic relationship between the human and chicken RHCE genes supports orthology between these genes.

## Discussion

Two GWAS, one with 600K SNPs and one with 54K SNPs, using samples from multiple independent sources detected the same genomic region associated with I blood system alleles. Further examination based on sequence information from inbred lines and samples with known serology showed that the only SNPs that fit with the I system serological segregation pattern were those within the *RHCE* gene. Genotype information from SNP assays within the *RHCE* gene and subsequent RHCE-haplotype identification showed consistent association of I serological allele with specific RHCE haplotypes. Repeated and consistent RHCE haplotype and chicken I blood system serological allelic associations from multiple independent sources validate that *RHCE* is the gene responsible for the chicken I blood system. While some inconsistencies were found, most of these can be attributed to failure of accurately distinguishing heterozygotes based on serology, which is likely due to weak serological reagents. Heterozygosity in serological reactions is identified by failure to hemagglutinate at more dilute antisera levels, which can be somewhat subjective, thus leading to misidentification. The observation that the same serological alleles were found for different haplotypes (i.e. I^8^ = RHCE-H01, H03, H04 and H10) can be due to the lack of antigenic differences among these haplotypes, or failure of the antisera to identify antigenic differences. Exchange of specific serological reagents between UK and US laboratories occurred in the mid 1950’s, but did not include the I system [48]. There is no record of subsequent cross laboratory comparisons with different laboratory antisera and fresh red blood cells to confirm identity of serological reagents, which can result in inconsistent identification of the alleles and could explain why the same haplotype (RHCE-H03) was found to be associated with both I^4^ and I^8^ (typing was done in different laboratories; UCD003 at UCD, WL2 and WL3 at NIU, RHC at Houghton UK).

The observation that several RHCE haplotypes show a deletion of a large part of the *RHCE* gene is intriguing. Most of these ‘short’ haplotypes were found at a very low frequency (< 1%) in the DNA samples of chicken populations available 20 + generations ago, and disappeared within three to four generations, which suggests a detrimental impact of the truncated RHCE protein. However, one of the lines (RIR1) has shown a steady increase in the frequency of the ‘short’ RHCE-H07haplotype from 3 to 35% across 19 generations. Since RIR1 is a highly selected elite egg laying line, the continued presence and relatively high frequency of this haplotype suggests that it does not confer any negative impact on production traits under selection. Indeed, this ‘short’ haplotype would have been eliminated from the breeding population if it had an undesirable effect on production, overall health, or livability. Equivalent RHCE deletion variants are rare in humans with the ‘D–phenotype’ being associated with the expression of RHD, but not RHCE. Two human variants have been reported to cause the D–phenotype, each with a partial deletion of the *RHCE* gene, i.e. one variant with a deletion of all the coding sequences of *RHCE* except for exon 1, and a second variant with a deletion of the coding sequences of exons 2 through 8 [49, 50]. While neither of these variants were reported to have an impact on health, these deletion variants can have significant impact on blood transfusion incompatibility [51].

Initial studies on genetic variation within the multiple chicken blood systems focused on the determination of the impact of these blood groups on phenotypic traits. Since the chicken MHC-B blood system was found to have such a profound impact on disease resistance [15, 16] early work focused on the other identified blood groups as potential genetic markers for important traits, particularly those related to immunology. The observation that divergently selected lines showed differences in I system allele frequencies [20–22] supported the value of blood systems as genetic markers for traits. However, little work had been done on non-B chicken blood groups over the past 40 years [15]. Identification of *RHCE* as the gene responsible for the chicken I blood system allows relevant information on *RHCE* gene function obtained in other species to be applied to the chicken. Furthermore, the chicken RHCE variants identified, particularly the deletion variants, could be an excellent model to understand the impact of RHCE deletion variants on various physiological parameters. The hypothesis that RHCE functions as a transmembrane CO_2_ transporter is intriguing and raises the question whether this gene has the same function in chickens and whether RHCE protein variants could have an impact on blood oxygen levels in chickens.

The use of genotyping to determine blood type phenotypes from DNA sequences has applications in human transfusion and transplantation medicine, as it can provide better information than serology alone and can be more cost-effective [52]. Identification of the gene responsible for the chicken I system and the subsequent development of PCR-based detection tests will allow reliable and rapid detection of I system variation for large sample numbers and perhaps determine whether variation of the I system has an impact on traits important for chicken health, welfare, and production.

## Conclusions

The identification of RHCE as the gene responsible for the chicken I blood system was based on samples from multiple independent sources with both serological information and DNA. Two independent GWAS defined the candidate genomic region, which led to subsequent SNP genotyping that confirmed the responsible gene. The observation of synteny between the chicken and human RHCE regions supports the homology of the genes. The identified RHCE deletion variants in viable birds suggests that these could be an excellent animal model for studying the impact of RHCE deletions on various physiological parameters, including health and productivity.

### Supplementary Information

Additional file 1: Table S1. *RHCE* comparison of antisera typing with *RHCE* SNP typing to identify chicken blood system I alleles. Cell color indicates agreement (green) or discrepancy (red) between genotypes tested with alloantisera vs SNPs.

## Data Availability

The data presented in this study are available on request from the corresponding author.
