# Supplementary material for: The RHCE gene encodes the chicken blood system I
Source: Genet Sel Evol. 2024 Jun 19;56:47. doi: 10.1186/s12711-024-00911-9 (PMC11188259; doi:10.1186/s12711-024-00911-9)
Supplement: Supplementary file 1 — Additional file 1: Table S1. RHCE comparison of antisera typing with RHCE SNP typing to identify chicken blood system I alleles. Cell color indicates agreement (green) or discrepancy (red) between genotypes tested with alloantisera vs SNPs. [file 12711_2024_911_MOESM1_ESM.docx]

**Table S1.** **Comparison of antisera typing with RHCE SNP typing to identify chicken blood system I alleles. Cell highlight indicates agreement (green) or discrepancy (red) between genotypes tested with alloantisera vs RHCE SNP haplotype.**

|  | **Serology type** | | |  |  |
| --- | --- | --- | --- | --- | --- |
| **Source** | **I^2^I^2^ (n)** | **I^2^I^8^ (n)** | **I^8^I^8^ (n)** | **SNP typing**  **correspondence** | **% correct** |
| **WL1** |  |  |  |  |  |
| SNP H01H01 | 0 | 2 | 14 | 14/16 |  |
| SNP H01H02 | 0 | 40 | 14 | 40/54 |  |
| SNP H02H02 | 19 | 0 | 0 | 19/19 |  |
| **Line total** |  |  |  | 73/89 | 82.0 |
| **WL2** |  |  |  |  |  |
| SNP H02H02 | 29 | 0 | 0 | 29/29 |  |
| SNP H02H03 | 0 | 38 | 1 | 38/39 |  |
| SNP H03H03 | 0 | 5 | 6 | 6/11 |  |
| **Line total** |  |  |  | 73/79 | 92.4 |
| **WL7** |  |  |  |  |  |
| SNP H01H01 | 0 | 4 | 15 | 15/19 |  |
| SNP H01H02 | 0 | 11 | 0 | 11/11 |  |
| SNP H02H02 | 4 | 0 | 0 | 4/4 |  |
| **Line total** |  |  |  | 30/34 | 88.2 |
| **NIU DNA bank pedigree** |  |  |  |  |  |
| SNP H02H02 | 21 | 1 | 0 | 21/22 |  |
| SNP H02H03 | 0 | 4 | 1 | 4/5 |  |
| SNP H02H04 | 0 | 5 | 2 | 5/7 |  |
| SNP H03H03 | 0 | 0 | 1 | 1/1 |  |
| SNP H04H04 | 0 | 0 | 5 | 5/5 |  |
| **Line total** |  |  |  | 36/40 | 90.0 |
| **NIU DNA bank non-pedigree** |  |  |  |  |  |
| SNP H01H02 | 0 | 9 | 2 | 9/11 |  |
| SNP H01H04 | 0 | 2 | 0 | 0/2 |  |
| SNP H02H02 | 15 | 0 | 0 | 15/15 |  |
| SNP H02H03 | 0 | 3 | 0 | 3/3 |  |
| SNP H02H04 | 1 | 14 | 0 | 14/15 |  |
| SNP H03H03 | 0 | 0 | 38 | 38/38 |  |
| SNP H04H04 | 0 | 4 | 0 | 0/4 |  |
| **Line total** |  |  |  | 79/88 | 89.7 |
|  |  |  |  |  |  |
| **antisera typing**  **correspondence** | 88/89 | 124/142 | 76/99 | 288/330 | 87.2 |

Green box indicates consistency between RHCE SNP haplotype and I system serological alleles

Red box indicates inconsistency in the results between RHCE SNP haplotype and I system serological alleles
